# Cortical Excitability and Interhemispheric Connectivity in Early Relapsing–Remitting Multiple Sclerosis Studied With TMS-EEG

**DOI:** 10.3389/fnins.2018.00393

**Published:** 2018-06-08

**Authors:** Carl M. Zipser, Isabella Premoli, Paolo Belardinelli, Nazareth Castellanos, Davide Rivolta, Tonio Heidegger, Florian Müller-Dahlhaus, Ulf Ziemann

**Affiliations:** ^1^Department of Neurology and Stroke, Hertie Institute for Clinical Brain Research, Eberhard Karls University of Tübingen, Tübingen, Germany; ^2^Nirakara: Instituto de Investigación y Formación en Ciencias Cognitivas, Madrid, Spain; ^3^Department of Education Science, Psychology and Communication Science, University of Bari Aldo Moro, Bari, Italy; ^4^Department of Neurology, Goethe University Frankfurt, Frankfurt am Main, Germany; ^5^Department of Psychiatry and Psychotherapy, University Medical Center Mainz, Mainz, Germany

**Keywords:** TMS-EEG, multiple sclerosis, relapsing–remitting, excitability, connectivity, transcranial magnetic stimulation

## Abstract

Evoked potentials (EPs) are well established in clinical practice for diagnosis and prognosis in multiple sclerosis (MS). However, their value is limited to the assessment of their respective functional systems. Here, we used transcranial magnetic stimulation (TMS) coupled with electroencephalography (TMS-EEG) to investigate cortical excitability and spatiotemporal dynamics of TMS-evoked neural activity in MS patients. Thirteen patients with early relapsing–remitting MS (RRMS) with a median Expanded Disability Status Scale (EDSS) of 1.0 (range 0–2.5) and 16 age- and gender-matched healthy controls received single-pulse TMS of left and right primary motor cortex (L-M1 and R-M1), respectively. Resting motor threshold for L-M1 and R-M1 was increased in MS patients. Latencies and amplitudes of N45, P70, N100, P180, and N280 TMS-evoked EEG potentials (TEPs) were not different between groups, except a significantly increased amplitude of the N280 TEP in the MS group, both for L-M1 and R-M1 stimulation. Interhemispheric signal propagation (ISP), estimated from the area under the curve of TEPs in the non-stimulated vs. stimulated M1, also did not differ between groups. In summary, findings show that ISP and TEPs were preserved in early-stage RRMS, except for an exaggerated N280 amplitude. Our findings indicate that TMS-EEG is feasible in testing excitability and connectivity in cortical neural networks in MS patients, complementary to conventional EPs. However, relevance and pathophysiological correlates of the enhanced N280 will need further study.

## Introduction

Multiple sclerosis (MS) is an inflammatory and secondary neurodegenerative disorder associated with disseminated cortical and white-matter lesions (Dendrou et al., 2015). Structural (i.e., MRI) and functional (i.e., evoked potentials, EPs) biomarkers of neurodegeneration in MS were proposed, that potentially can monitor treatment effects of disease-modifying drugs (Ziemann et al., 2011; Hardmeier et al., 2017).

Recording of motor EPs (MEPs) obtained with transcranial magnetic stimulation (TMS) can be used to measure the excitability of the human motor cortex as well as the functional integrity of the corticospinal tract and callosal fibers (Kobayashi and Pascual-Leone, 2003; Jung et al., 2006; Ziemann, 2011). Given these properties, TMS has been applied in the context of MS disease to assist diagnostic processes. Results show that low MEP amplitudes and slow central motor conduction time (CMCT) reflect axonal loss or chronodispersion due to inhomogeneous conduction slowing in fibers of the corticospinal tract (Hess et al., 1986, 1987).

A multimodal EP examination including MEPs, visual EPs (VEPs), and somatosensory EPs (SEPs) is established for detecting subclinical lesions in their respective functional system and might facilitate diagnosis of MS at an early disease stage (Ziemann et al., 2011). These EPs furthermore correlate with clinical disability in the respective functional system (Leocani et al., 2006; Chen et al., 2008). Multimodal EPs have also been demonstrated to predict disability progression in early relapsing–remitting MS (RRMS) (Jung et al., 2008).

With respect to transcallosal fibers, paired-coil TMS demonstrated malfunctioning of short-interval interhemispheric inhibition (sIHI) in early MS patients (Wahl et al., 2011). In addition, diffusion-tensor MRI revealed reduced fractional anisotropy (FA) in motor callosal fibers indicating microstructural damage. Importantly, healthy control (HC) subjects showed a significant linear correlation between sIHI and FA that was absent in the MS patients, suggesting that reduced FA in MS accounted for impaired sIHI (Wahl et al., 2011).

Transcranial magnetic stimulation coupled with electroencephalography (TMS-EEG) allows a *direct* investigation of cortical excitability and causal connectivity between different cortical areas (Cracco et al., 1989; Ilmoniemi et al., 1997; Ziemann et al., 2011; Rogasch and Fitzgerald, 2013; Hallett et al., 2017). TMS of the primary motor cortex evokes a complex EEG response with a specific spatiotemporal activation pattern across both hemispheres. The TMS-evoked EEG potentials (TEPs) consist of a sequence of positive and negative deflections at specific and reproducible peak latencies (P25, N45, P70, N100, P180, and N280) (Ilmoniemi et al., 1997; Komssi et al., 2004; Bonato et al., 2006; Lioumis et al., 2009). A recent study showed that the TMS-evoked signal propagates from the stimulated cortical area to the contralateral hemisphere using the corpus callosum (CC) as anatomical structure (Voineskos et al., 2010). Thus, TMS-EEG offers a unique opportunity to non-invasively assess intra- and interhemispheric neural signal propagation and the application of TMS-EEG to investigate impaired cortical excitability and connectivity in MS population seems rather straightforward. This approach has been successfully taken to enhance our understanding of the pathophysiology of several neuropsychiatric disorders, such as epilepsy (Valentin et al., 2008; Shafi et al., 2015), Alzheimer’s disease (Ferreri et al., 2016), schizophrenia (Noda et al., 2018), and attention-deficit hyperactivity disorder (Bender et al., 2005), whereas to date it has not been applied in MS.

Given microstructural alterations in the CC of early-stage MS and evidence that TEPs may propagate interhemispherically via the CC, we hypothesize that TEP features (i.e., amplitude, latency, interhemispheric propagation) would differ between MS patients and HCs. Specifically, we expect changes in TEP amplitudes underlying altered cortical excitability and a delay in TEP propagation from the stimulated area (M1) to interconnected remote brain areas. We here aim to provide first insights of TMS-EEG application in patients with RRMS.

To this end, TMS-EEG was conducted in 13 patients with RRMS and 16 matched HCs. TEPs were elicited by stimulation of left and right primary motor cortex (L-M1 and R-M1), respectively. The amplitudes and latencies of the typical TEP components were compared between the two groups by means of a cluster-based analysis suited for this purpose (Maris and Oostenveld, 2007), and an interhemispheric propagation analysis was used to investigate possible alterations in cortical connectivity due to CC lesions.

## Materials and Methods

### Subjects

Thirteen MS patients (mean age 37.0 ± 8.0 years, range 22–48 years, nine females) and 16 age- and gender-matched healthy volunteers (HCs; mean age, 30.0 ± 8.0 years, range 23–49 years, eight females) participated in the study after giving their written informed consent (**Table 1**). MS patients were diagnosed according to the revised McDonald criteria for relapsing–remitting type (Polman et al., 2011), and only those with a score <3 on the Expanded Disability Status Scale (EDSS) (Kurtzke, 1983) were included in the study. Relapse or glucocorticoid treatment within 3 months prior to participation in this study was an exclusion criterion for enrollment. Median EDSS was 1.0 (range 0–2.5) and median disease duration was 12 months (range 3–144 months). Patient characteristics including current pharmacological treatment are summarized in **Table 1**. All subjects were right-handed according to the Edinburgh Handedness Inventory (laterality score ≥75%) (Oldfield, 1971). None of the participants had any contraindication to TMS, or any neurological, psychiatric, or other relevant medical diseases other than MS (Rossi et al., 2011; Groppa et al., 2012). Further exclusion criteria were (i) intake of CNS active drugs within the last 3 months, (ii) abuse of drugs (including alcohol and nicotine), and (iii) pregnancy. The study protocol conformed to the latest revision of the Declaration of Helsinki and was approved by the local Ethics Committee of the Hospital of the Medical Faculty of Goethe University Frankfurt.

**Table 1:** Patient characteristics.

Patient #	Disease duration	EDSS	MRI	Medication	RMT		MEP	SEP	VEP	AEP
					L	R				
1	4	1	PV, IT	None	48	48	P	N	P	N
2	8	0	PV, IT, CC	Copaxone	42	44	N	N	P	N
3	10	1.5	PV, JC, CC	None	62	64	N	N	P	N
4	144	2.5	N.A.	Copaxone	58	59				
5	26	1	N.A.	Copaxone	54	60				
6	84	1	N.A.	None	64	54				
7	54	0	PV, CC, ON	Copaxone	58	64	N	N	P	N
8	4	0	PV, CC, IT	IF-beta	63	63	N	N	P	N
9	12	1	PV, IT	IF-beta	65	65	P	P	P	N
10	3	2	N.A.	IF-beta	46	46				
11	7	1.5	PV, JC, IT	IF-beta	56	61				
12	20	0	PV, CC, IT	Copaxone	57	48	P			
13	72	1	PV, CC	IF-beta	50	50				

*#, patient number. Disease duration in months. EDSS, Expanded Disability Status Scale. The column MRI provides information on the localization of T2w hyperintense MS-lesions in latest MRI available (CC, corpus callosum; IT, infratentorial; JC, juxtacortical; ON, optic nerve; PV, periventricular). Of note, MRI and evoked potentials were not a prerequisite for study inclusion and thus are not available [n.a.] for analysis in some patients. Medication indicates current medication (IF-beta: interferon-beta). RMT: resting motor threshold in maximum stimulator output (MSO%) for left (*L*) and right (*R*) primary motor cortex. Evoked potentials: P, pathological, N, normal finding. Blank indicates not available. MEP: motor evoked potentials (upper/lower extremities); SEP: somatosensory evoked potentials (upper/lower extremities); VEP, visual evoked potentials; AEP, auditory evoked potentials. Evoked potentials were recorded, then analyzed and evaluated in line with the current International Federation of Clinical Neurophysiology (IFCN) guidelines (Celesia et al., 1993; Rossini et al., 1994, 2015; Cruccu et al., 2008; Robson et al., 2018). Normal and pathological findings were discriminated further according to hospital- and device-specific normative data*.

### Study Design

**FIGURE 1:**
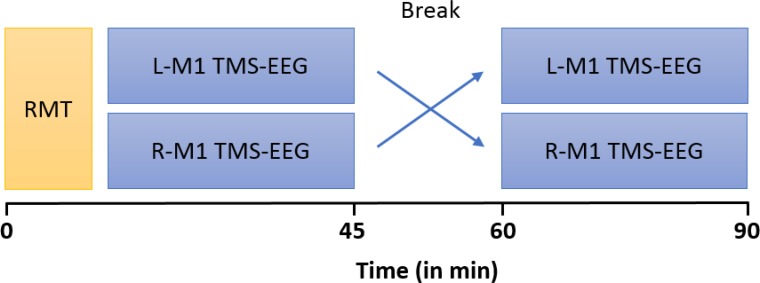
Timeline of experiments. MS patients and HCs underwent two blocks of stimulation which consisted of 150 single TMS pulses at an intensity of 100% RMT each, over left (L) and right (R) M1, respectively. RMT of L-M1 and R-M1 were first measured in randomized order and subsequently the TMS-EEG trials were performed.

Participants were seated in a comfortable reclining chair, with eyes open and fixated a marked cross on the wall in front of them. During the stimulation protocol, subjects were instructed to keep the right (or left) *abductor pollicis brevis* muscle (APB) relaxed while EMG was continuously monitored on a computer screen. A total of 150 single TMS pulses were applied to the L-M1 and R-M1, respectively with a 15 min break between stimulation epochs (**Figure 1**). Stimulation of L-M1 and R-M1 was randomized across subjects.

### Transcranial Magnetic Stimulation

Focal TMS of the hand area of L-M1 and R-M1 were delivered through a Magstim-200 stimulator connected to a figure-of-eight coil with external loop diameters of 90 mm (Magstim Company, Carmarthenshire, United Kingdom) with a monophasic current waveform. The coil was held tangentially to the skull with the handle pointing backward and laterally at a 45° angle to the sagittal plane. The optimal coil positions over the hand area of L-M1 and R-M1 for eliciting MEPs in the right APB, and the left APB respectively, were determined as the site where TMS at a slightly suprathreshold intensity consistently produced the largest MEP amplitudes. These coil positions of the APB hotspots in the L-M1 and R-M1 were marked with a felt pen on the EEG cap to ensure constant coil placement throughout the experiment. MEPs were recorded with Ag–AgCl surface cup electrodes in a belly-tendon montage. The EMG signal was recorded by a D360 amplifier (Digitimer, Hertfordshire, United Kingdom) and band-pass filtered from 20 Hz to 2 kHz, digitalized via a CED 1401 I/O board (Cambridge Electronic Design, Cambridge, United Kingdom) at an A/D rate of 10 kHz per channel and displayed online. The resting motor threshold (RMT) was determined using the relative frequency method (Groppa et al., 2012) to the nearest 1% of maximum stimulator output (MSO). It was defined as the minimum intensity that was sufficient to elicit an MEP of >50 μV peak-to-peak amplitude in at least 5 out of 10 subsequent trials. RMT is reported as percentage of MSO.

### High-Density EEG Recording During TMS

A TMS-compatible EEG system (BrainAmp DC, Brain Products GmbH, Munich, Germany), which prevents amplifier saturation and allows continuous EEG recording during TMS delivery, was used to measure TEPs. The EEG signal was digitized at a sampling frequency of 5 kHz and continuously recorded from 62 electrodes mounted on an elastic cap according to the standard international 10-20 EEG system (BrainCap-Fast’n Easy, Brain Products GmbH). In addition, eye movements were recorded by placing an electrode over the outer canthus of the left eye and an electrode placed below the right eye. The impedance of all electrodes was kept <5 kΩ throughout the experiment.

In order to minimize the TMS induced artifact, the EEG electrode wires were rearranged radially away from the fixed coil position (Sekiguchi et al., 2011). Further, during stimulation a masking sound was played through ear phones to minimize an auditory EP induced by the coil click (Nikouline et al., 1999; Massimini et al., 2005). The volume of the masking noise (always below 90 dB) was progressively adjusted until the subjects reported that the TMS click was no longer perceptible. The sound was then maintained constant across stimulation sessions and was interrupted during the break between stimulation epochs. L-M1 and R-M1 were stimulated in a randomized order by applying two blocks of 150 TMS pulses each at 100% RMT intensity, every 5 s with a random variation of 25% to reduce anticipation of the next trial. At this stimulus intensity (SI), no MEP or only miniature MEPs are elicited by the TMS pulse. Thus, although unlikely, we cannot completely exclude that TEPs were contaminated by somatosensory afferent signals from muscle twitches, as assessed by recent studies (Fecchio et al., 2017; Petrichella et al., 2017).

### Data Processing and Analysis

Analysis of EEG data was performed using the Fieldtrip open source Matlab toolbox^1^ (Oostenveld et al., 2011) and custom-made scripts following a multistep procedure (Premoli et al., 2014a,b). EEG data were first referenced to the linked mastoids (channels TP9 and TP10) and down-sampled to 1000 Hz. EEG trials containing large artifacts, eye movements, or muscle activation were then visually detected and discarded from further analysis. Every trial was detrended and band pass filtered between 2 and 80 Hz. A notch filter (50 Hz) was applied to reduce line noise contamination. EEG trials were then segmented on the continuously recorded EEG time series from -500 ms before to 500 ms after TMS pulses. The TMS artifact was removed by applying a linear interpolation for a 10 ms interval before and after the TMS pulse (Thut et al., 2011). The remaining TMS-EEG trials for the MS group (L-M1, 132 ± 11 trials, R-M1 136 ± 10 trials) and the control group (L-M1 131 ± 10 trials, R-M1 131 ± 11 trials) were then baseline corrected in the period from -500 to -15 ms before TMS. TEPs were calculated by averaging the EEG signal over all retained trials for each channel. To smooth the TEP, a band-pass filter from 1 to 45 Hz was applied. According to previous literature (Lioumis et al., 2009) TEP components (P, positive deflection; N, negative deflection) were identified in distinct, non-overlapping time windows of interest (TOI) and considered for further analysis: N45 (TOI: 30–60 ms), P70 (TOI: 60–80 ms), N100 (TOI: 80–150 ms), P180 (TOI: 151–250 ms), and N280 (TOI: 251–350 ms). TOIs were chosen based on grand-averages of TEPs, separately for the MS and HC group. Early components (i.e., N15/P30) were contaminated by the TMS artifact and not included in the current analysis. We tested significant differences between the two groups by applying independent *t*-tests for TEP (i) amplitudes and (ii) latencies in their specified TOIs. The cluster-based permutation methodology for amplitudes is well explained in (Maris and Oostenveld, 2007) and in our previous work (Premoli et al., 2014a). We have used the same approach to overcome an arbitrary preselection of regions of interest to compare TEP latencies. To this end, from a structure composed by channels and time points within each TOI, for each subject we extracted the latency value corresponding to the maximum value of absolute amplitude (considering the possibility to have both positive and negative peaks). The resulting 3-D matrix, with dimensions (1) subjects, (2) channels, and (3) extracted latency values was used within the framework of the cluster-based permutation analysis.

The propagation of TMS-induced cortical EPs between the motor cortices was estimated in MS patients and HCs by means of interhemispheric signal propagation (ISP) (Voineskos et al., 2010). The averaged single-trial rectified TEP curves from the C3 (closest electrode to M1 hand knob in the left hemisphere) and C4 (closest to the R-M1 hand knob) electrodes were calculated. Depending on the stimulated M1, we considered as periods for the area under the curve (AUC) the time samples between 50 and 150 ms (stimulated M1) and between 60 and 160 ms (non-stimulated M1). For the stimulated M1, the time-window of 50–150 ms was chosen with respect to the onset of artifact-free recordings. For the non-stimulated M1, the shift by 10 ms assumed an ISP time through the CC of 10 ms (Ferbert et al., 1992; Meyer et al., 1995; Komssi et al., 2002). Then the ISP was calculated as the percentage of AUC (non-stimulated M1)/AUC (stimulated M1) (Voineskos et al., 2010).

### Statistical Analysis

Mean RMT and standard error of the mean (SEM) were calculated. RMT was compared between MS patients and HC using a two-tailed Student’s *t*-test for unpaired samples, and within-group comparisons between L-M1 and R-M1 stimulation were evaluated using two-tailed paired Student’s *t*-tests (statistical significance assumed at *p* < 0.05).

Significant differences between MS patients and HC in TEP latencies and amplitudes were assessed using independent samples *t*-tests. To correct for multiple comparisons (i.e., electrodes, time points) we conducted a non-parametric cluster-based permutation analysis as implemented in fieldtrip (Maris and Oostenveld, 2007). Statistical tests were computed separately for the analysis of amplitudes and latencies for each TOI in all the electrodes, separately for L-M1 and R-M1 stimulation, and in line with our previous work (Premoli et al., 2014a).

Two-tailed, Student’s *t*-tests for unpaired samples were used to test whether the left-to-right M1 and right-to-left M1 ISP were altered in MS patients compared to HC.

## Results

All participants complied with the study protocol and no adverse effects were reported.

### Motor Cortical Excitability

We found a group difference showing overall higher RMT values in MS than in HC. However, we found no main effect of site of stimulation, and no group by site interaction. Mean RMT of L-M1 was 49 ± 6 %MSO in HC vs. 56 ± 7 %MSO in MS. For R-M1, mean RMT was 50 ± 7 %MSO in HC vs. 56 ± 8 %MSO in MS.

There was no difference of RMT between L-M1 and R-M1 in MS [*t*(12) = -0.170, *p* = 0.868], whereas HC had a lower RMT in L-M1 vs. R-M1 [*t*(15) = -2.192, *p* = 0.045]. Moreover, RMT was higher in MS patients than in HC in both L-M1 [*t*(12) = 3.525, *p* = 0.004] and R-M1 [*t*(12) = 2.561, *p* = 0.025].

### Grand-Averaged TEPs After L-M1 and R-M1 Stimulation

**FIGURE 2:**
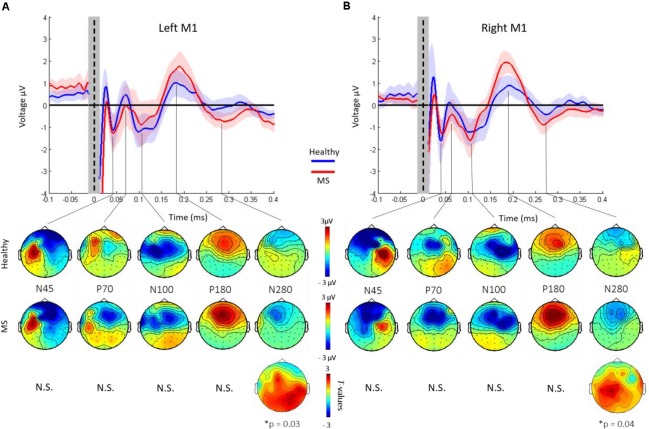
Grand-average TEPs across all channels of healthy controls (blue) and MS patients. Shades represent ±1 SEM. TEPs are shown for left **(A)** and right **(B)** M1 stimulation, respectively. Vertical dashed line, time of TMS. The shaded gray bar represents the part of the EEG trace (±10 ms) that was linearly interpolated to remove the TMS-induced artifact. TEP components are labeled according to their polarity and approximate latency. Lower panel shows topographical distributions of surface voltages (μV) for the most pronounced TEP components (N45, P70, N100, P180, and N280), for healthy controls (upper row) and MS patients (lower row), respectively. The N280 amplitude was significantly larger in the MS cohort compared to HCs (*p* < 0.05). Bottom row: *t*-statistic maps of the TEP amplitude differences (HC minus MS). Black dots indicate significant channels, predominantly in the non-stimulated hemisphere.

Grand-average TEPs after L-M1 and R-M1 stimulation showed the well-known TEP pattern with the most reproducible peaks N45, P70, N100, P180, and N280 (**Figure 2**) and topographical voltage distribution in line with previous TMS-EEG studies (Komssi et al., 2004; Premoli et al., 2014a; Darmani et al., 2016). Cluster-based permutation analysis revealed no statistically significant differences in the latency of the N45, P70, N100, P180, and N280 components (all *p* > 0.05). In terms of amplitudes, there were no significant differences for the N45, P70, N100, and P180 components (all *p* > 0.05). In contrast, the N280 component was significantly larger in the MS vs. the HC group, both for L-M1 and R-M1 stimulation (for L-M1 *p* = 0.03, for R-M1 *p* = 0.04; **Figures 2A,B**, lower panels). The topography of significant differences in N280 amplitude between the MS and HC group was primarily located in the non-stimulated hemisphere (**Figures 2A,B**, lower panels).

**FIGURE 3:**
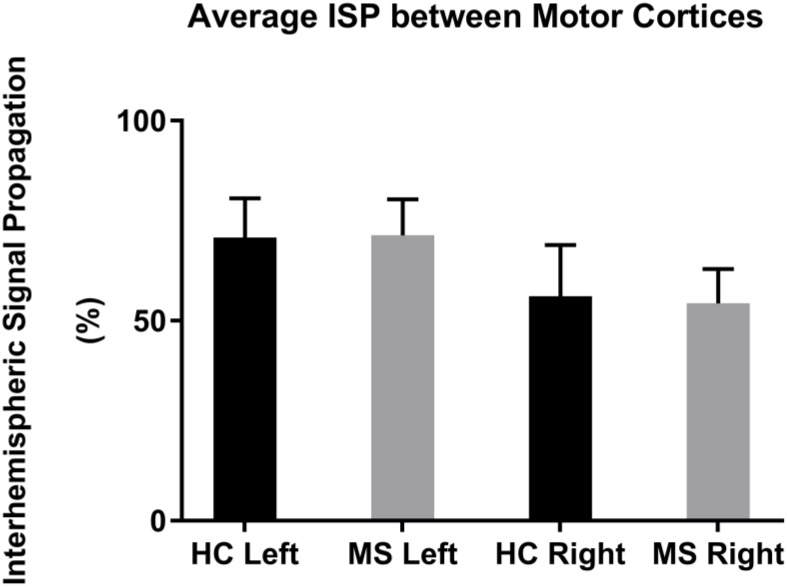
Mean ISPs in healthy controls (HC) and MS patients for left and right motor cortex stimulation. Error bars represent 1 SEM.

The mean left-to-right M1 ISP was 70.8 ± 10.0% (mean ± 1 SEM) in HC and 71.4 ± 9.0% in MS. The mean right-to-left M1 ISP was 56.1 ± 12.8% in HC and 54.4 ± 8.6% in MS patients (**Figure 3**). Both, the left-to-right M1 ISP and right-to-left M1 ISP did not show a significant difference between HC and MS [*t*(27) = -0.41, *p* > 0.05; *t*(27) = 0.11, *p* > 0.05, respectively].

## Discussion

Here we used TMS-EEG to assess cortical excitability and connectivity in early RRMS. Results did not show signal propagation delay as we found similar TEP latencies in MS patients vs. HCs and no differences in TMS-evoked activity between hemispheres in central electrodes. In contrast, the N280 TEP amplitude was significantly larger in the MS- vs. the HC group, with no amplitude differences in earlier TEP components between groups. RMT was higher in MS patients, both for L-M1 and R-M1 stimulation with no hemispheric asymmetries present. Conversely, RMT in HC was higher in the non-dominant right-hemisphere. Finally, neither group has shown hemispheric asymmetries in terms of TEPs.

### Cortical Excitability: Motor Threshold in Multiple Sclerosis

Resting motor threshold is assumed to reflect neural excitability, in particular excitability of axons of long-range cortico-cortical neurons, regulated by voltage-gated sodium channels, and their fast ionotropic glutamatergic synapses onto corticospinal neurons (Ziemann et al., 2015). Demyelination and neurodegeneration in MS might lead to abnormalities of cortical excitability (Vucic et al., 2011). As CMCT was normal in the majority of our cases (**Table 1**), the increase in RMT may have predominantly originated in cortical pathology, as previously suggested (Caramia et al., 1991).

In this study, RMT was higher in the MS patients for both the L-M1 and R-M1. Previous studies on RMT in MS populations have shown inconsistent results: Some studies did not observe higher RMT in the relapsing–remitting subtype of MS (RRMS), but only in secondary progressive forms (Conte et al., 2009; Vucic et al., 2011), whereas others have demonstrated higher RMT in RRMS (Liepert et al., 2005; Nantes et al., 2016; Neva et al., 2016). The patient groups of these studies were similar for patients’ characteristics (i.e., age and disability) and sample sizes to our cohort. Additionally, TMS excitability measures might be influenced through gender-specific neuro-hormonal changes (Smith et al., 1999). Our experiments were controlled for circadian rhythm: all subjects were examined around the same time of day (8–12 a.m.); shift-workers were excluded to avoid examination-time bias (Chellappa et al., 2016).

### TMS-Evoked EEG Potentials in Multiple Sclerosis and Healthy Controls

Transcranial magnetic stimulation coupled with EEG is continuously evolving toward a powerful tool to obtain direct information about human cortical excitability and connectivity. Several TMS-EEG studies showed robustness of TEPs in terms of their spatiotemporal profile, highlighting their possible development as biomarkers of human cortical functions (Hallett et al., 2017).

In contrast to our expectation of an abnormal TEP pattern in MS, the single abnormality was a larger amplitude of the N280 after stimulation of both, the L-M1 and R-M1. The nature of this TEP component is not yet well understood. However, previous studies associated the long latency and wide topographical distribution with the engagement of reverberant cortico-subcortical circuits (Ferreri et al., 2011), similar to the P180. To further support this notion, TMS-EEG investigations of deep sleep stages, drug-induced anesthesia and disorders of consciousness showed a complete suppression of TMS-evoked activity >150 ms post-stimulus together with a breakdown of signal propagation to brain areas distant from the site of stimulation (Massimini et al., 2005; Ferrarelli et al., 2010; Rosanova et al., 2012; Sarasso et al., 2015). In addition, TMS-EEG studies in disorders of increased cortical excitability, such as epilepsy, showed enlarged amplitudes of late TEPs (Shafi et al., 2015; Ter Braack et al., 2016). Therefore, despite lack of information about the exact neurophysiological underpinnings of the N280 TEP component, our results are in line with the notion that the increased N280 TEP amplitude in MS patients reflects altered long-range cortical excitability and connectivity.

None of the earlier TEP components showed significant abnormalities in the MS group. TMS paired-pulse and pharmacological studies suggested, that the early TEPs (N15-P30, not analyzed in our study) likely reflect cortical excitation at the site of stimulation (Paus et al., 2001; Esser et al., 2006), while later TEPs (N45–N100) are linked to fast and slow GABAergic inhibitory neurotransmission processes (Premoli et al., 2014a; Darmani et al., 2016). Our nil findings may have several explanations. Firstly, the lesion load in the CC in our early-stage MS group may not have been severe enough to interfere with inter-hemispheric TEP propagation. Secondly, TEP components are thought to reflect distributed circuit properties involving diverse cortico-cortical and cortico-subcortical-cortical connections (Rosanova et al., 2009) that may have the capacity to maintain effective connectivity as expressed by TEPs despite the presence of localized structural lesions.

Importantly, TEPs earlier than the N45 (i.e., N7/P13/ N18/P30) could not be investigated due to contamination of the EEG signal by the TMS artifact. Since the activation of the non-stimulated hemisphere occurs within the first 12–25 ms (Ferbert et al., 1992; Meyer et al., 1995; Komssi et al., 2002), abnormalities in TEP propagation might be revealed only in this early post-stimulus interval. This will need further investigation in future studies.

### ISP in Multiple Sclerosis and Healthy Controls

The statistical comparison of ISP of MS patients vs. HCs did not result in significant differences in the L-M1 or R-M1 stimulation conditions. MS patients showed a slightly larger ISP in the left-to-right M1 condition. This result was reversed in the right-to-left M1 condition (HC showing a slightly larger mean than MS; **Figure 3**). Voineskos et al. (2010) found an inverse correlation between ISP and functional anisotropy of callosal fibers, highlighting a possible inhibitory role for callosal fibers with suprathreshold stimulation. In our data, we could not find larger ISPs in the MS group, possibly due to impaired callosal inhibitory mechanisms, as previously observed in MS patients at an early stage (Wahl et al., 2011, 2016).

### Cortical Asymmetries in RMT and TEPs

Our findings provide evidence that markers for cortical and interhemispheric network properties, expressed through TEP amplitude/latency and RMT, did not reflect cortical asymmetries in early-stage MS. This finding was not related to handedness, since all participants were right-handed. Applying TMS in randomized order to left and right hemisphere prevented bias through habituation.

Motor threshold in healthy volunteers was lower in the dominant motor cortical hemisphere, as previously reported in right-handers (De Gennaro et al., 2004; Ilic et al., 2004). Others have not observed these differences (Civardi et al., 2000). Those conflicting results might be related to confounding factors mentioned earlier. In terms of TEPs, one prior study systematically compared single pulse M1 stimulation between hemispheres, but did not find any hemispheric differences (Lioumis et al., 2009). In MS patients, a comparable cohort has shown functional hemispheric asymmetries, expressed through prolonged MEP latencies in the dominant (left) hemisphere (Neva et al., 2016). Since MEP latencies mostly reflect integrity of the corticospinal tract, this does not help in understanding intracortical hemispheric asymmetries. A proper literature comparison of RMT laterality in MS was difficult since previous studies were constrained to testing of the dominant hemisphere.

### Study Limitations

Some limitations to this study need to be mentioned. First, the statistical power might be insufficient due to low number of patients enrolled in the study. The sample size was estimated based on previous TMS-EEG/-EMG studies in HC and MS, which aimed to evaluate cortical excitability and interhemispheric conduction (Lioumis et al., 2009; Wahl et al., 2011).

Various disease-modifying drugs in MS might have affected the results. For beta-interferons it has been demonstrated that MEP abnormalities might be improved within months during therapy (Feuillet et al., 2007). In contrast, there is no evidence for relevant changes in EPs through glatiramer acetate (Ehling et al., 2015). Most importantly, patients with recent glucocorticoid-therapy were excluded since changes of cortical excitability have been demonstrated under steroids within days (Ayache et al., 2014).

In terms of structural correlation, this study was limited since imaging data were not obtained in all patients, and microstructural assessment using diffusion-tensor imaging was not done in any of the patients. In the early stages of MS, interhemispheric fiber tracts have shown to suffer from structural lesions, correlating with functional impairment (Bester et al., 2008; Wahl et al., 2011). It might also be objected, that TMS was not MR-navigated. However, this was not necessary because the motor hotspot was defined with TMS-EMG and monitored throughout the measurements.

Another relevant issue to be addressed is the elevated SI in MS group compared to HC group caused by the difference in RMT. We assumed that SI did not bias TEP amplitudes in the MS cohort. It was demonstrated previously, that size of TEP amplitudes P30–P180 depended on SI, increasing in a non-linear (N15–N45) or in a linear fashion (N100 and P180) (Komssi et al., 2004). The only TEP amplitude to be increased in MS was the N280, which has not been investigated in terms of SI-dependency previously. However, there was no rationale for an isolated increased N280 through elevated SI. Hence, we believe that the increased N280 amplitude in the MS group was not caused by the SI group difference.

## Conclusion

As per our understanding of MS as a disease characterized by demyelination and axonal degeneration, we sought to explore pathological circuits and evaluate brain excitability with TMS-EEG. In conclusion, from our sample group, we showed that it is feasible to record and quantify TEPs in RRMS patients, which, compared to healthy volunteers, showed similar features except for a larger N280 component. Notably, there was no clear evidence for altered ISP. Given our limited knowledge of the physiology of the N280 further research is needed, e.g., pharmacological TMS-EEG studies, and correlation with behavioral data. To conclude, findings suggest that TMS-EEG is a promising technique, although more studies are needed to evaluate the relevance in clinical populations.

## Author Contributions

CZ contributed to data acquisition, data analysis, and manuscript writing. IP contributed to data analysis and manuscript writing. NC, IP, PB, and DR contributed to data analysis. TH contributed to data acquisition and manuscript writing. FM-D contributed to data discussion and manuscript writing. UZ contributed to experimental design, data analysis, and revision of the manuscript.

## Conflict of Interest Statement

The authors declare that the research was conducted in the absence of any commercial or financial relationships that could be construed as a potential conflict of interest.

## Footnotes

1 www.ru.nl/fcdonders/fieldtrip/
